# Acknowledgement to Reviewers of *Entropy* in 2017

**DOI:** 10.3390/e20010066

**Published:** 2018-01-15

**Authors:** Entropy Editorial Office

**Affiliations:** MDPI AG, St. Alban-Anlage 66, 4052 Basel, Switzerland

Peer review is an essential part in the publication process, ensuring that Entropy maintains high quality standards for its published papers. In 2017, a total of 684 papers were published in the journal. Thanks to the cooperation of our reviewers, the median time to first decision was 26.98 days and the median time to publication was 53.5 days. The editors would like to express their sincere gratitude to the following reviewers for their time and dedication in 2017:
Abah, ObinnaLi, ZhixiongAbbas, AliLian, WenzhaoAbbas, Ali E.Liang, ChaoAbbot, MaysamLiang, Chiu-kuoAbd-Alhameed, RaedLiao, BolinAbdAllah, Eslam G.Lieb, ElliottAboalayon, KhaldLIEBMAN, Joel F.Abrams, CameronLiemert, AndreAbutayeh, MohammadLietman, Thomas MAcedo, LuisLima, J.A.S.Acharya, U RajendraLima, MarkusAdams, StefanLin, Chih-HongAdelantado, FerranLin, HongAdesso, GerardoLin, JunlinAfonso, ClitoLin, Pei-FengAgop, MarcielLin, Shih-ChunAguiar, MarcusLin, Yu-JauAguilar, CristinaLinn, Jimmy B.Aguirre-Salado, Carlos A.Lio, YuhlongAhmad, FayyazLipniacki, TomaszAhmadi, MehdiLipowski, AdamAhmadi, PouriaLiu, ChongxinAhmadlouydarab, MajidLiu, ChunhuaAhmed, Mosabber UddinLiu, FangAhn, Choon KiLiu, Jain-ShingAjgl, JiříLiva, GianluigiAkbar, MuhammadLivadiotis, GeorgeAkbarzadeh, HamidLizier, JosephAkih-Kumgeh, BenLjubenko, AndrejAktaruzzaman, MdLlopis, RodrigoAkyol, EmrahLo Franco, Rosario LoAla-Nissilä, TapioLoaiciga, Hugo A.Al-Ashwal, Waddah A.Logsdon, Sally D.Alberto, Perea MorenoLoiola, Murilo BellezoniAlcaine, AlejandroLombardi, OlimpiaAlconero, Patricia LuisLombardo, Maria CarmelaAlexeev, AlexanderLong, GuiluAlfonso, LeonardoLongo, SandroAli, Sean A.Lopes, AntónioAlmgren, KhaledLopes, Artur O.Alonso, DanielLópez-Presa, José LuisAlonso, Joan FLópez-Ruiz, RicardoAlonso, Miguel A.Lorenz, ChrisAlonso-Serrano, AnaLorenz, Ralph D.Alvarez-Alonso, AntonioLosada, MarceloAmado, MiguelLostado, RubenAmaguchi, HideoLu, JianfengAmari, Shun-ichiLu, John C.-C.Amigó, JoséLu, RongxingAmiri, MehdiLuca, AdrianAmmari, HabibLucas, GaleAmmon, MartinLucia, UmbertoAmza, CatalinLuengo, DavidAndaluz, JoaquínLuger, George F.Anderson, Amy D.Lunev, Artem VAndo, TadashiLunghi, AlessandroAneziris, OlgaLunin, OlegAngstmann, ChristopherLuo, ChaominAngulo, Juan C.Luo, Jia-NingAngulo-Brown, FLuo, JianwenAnnila, ArtoLuo, ShunlongAnselmet, FabienLuongo, OrlandoAntal, PeterLupo, CosmoAntonopoulos, AngelosLux, ThomasAntuchevičienė, JurgitaLv, SongjunApfelbaum, E. M.Ly, AlexanderApiecionek, ŁukaszLyashenko, EugeniaAppadoo, Srimantoorao S.Lyra, MarceloAprea, CiroLyubushin, A. A.Arce, Pedro F.Lyuu, Yuh-dauhArcher, ReginaldMa, Andy JinhuaArdenghi, Juan SebastiánMa, TinghuaiArecchi, Fortunato TitoMa, XiaoliArgyros, IoannisMacedo, PedroArima, TakashiMachado, TenreiroArizmendi, MiguelMachta, JonathanArndt, MarkusMaciejewski, Andrzej JAros, RodrigoMackiewicz, MichalArroyo, DavidMacLellan, MichaelArsenić, IlijaMady, Carlos E.k.Arthanari, TiruMagenes, GiovanniArtiemjew, PiotrMahdavi, MahboobeAryal, JagannathMahian, OmidAsad, JihadMaina, Fadji ZaounaAsadi, BehzadMaiorino, AngeloAsboth, Janos K.Makse, Hernán A.Ashlock, DanMalakhov, DmitriAshton, NeilMalgaretti, PaoloAssar, SaïdMalvoni, MariaAssecondi, SaraMammone, NadiaAsselmeyer-Maluga, TorstenMancini, StefanoAstaras, AlexandrosMandal, AbhijitAste, AndreasMandic, DaniloAtangana, AbdonMangili, FrancescaAtoche, Alejandro CastilloManis, GeorgiosAubry, AugustoManiu, SilviuAurell, ErikMan'ko, VladimirAusloos, MarcelMann, Richard P.Awad, Ali IsmailMann, RobertAwan, ZohaibMann, Robert B.Awrejcewicz, JanManning, Gerald S.Ay, NihatMantzaris, Alexander V.Ayele, Yonas ZewduManzanares, José AntonioAzami, HamedMarcelli, ThierryBaaj, HassanMarcellin, Michael W.Baaliña Insua, ÁlvaroMarcolli, MatildeBaas, AndreasMarcon, EricBadescu, ViorelMarconi, Umberto Marini BettoloBadin, GualtieroMarek-Crnjac, LeilaBagnato, LucaMaren, Alianna J.Baillie, Richard T.Mari, AndreaBalasis, GeorgiosMarijuán, PedroBaldini, GianmarcoMarinazzo, DanieleBalduzzi, DavidMarinescu, CatalinaBaleanu, DumitruMarini, GustavoBalieu, RomainMarković, DimitrijeBamba, KazuharuMarmelat, VivienBan, Adrian I.Marmur, AbrahamBan, MasashiMarozas, VaidotasBandt, ChristophMarquet, PabloBanik, Suman KMarriott, PaulBañuls, Mari CarmenMarriott, Paul K.Barato, Andre C.Marsland, StephenBaraviera, Alexandre TavaresMartalo, MarcoBarberis-Blostein, PabloMartalò, MarcoBarbosa, Ramiro S.Martín, Manuel Jesús CoboBargh, Mortaza ShoaeMartinez, Ramses V.Bariviera, Aurelio F.Martínez-Rodrigo, ArturoBariviera, Aurelio FernándezMartinez-Valdes, EduardoBarkley, DwightMartinopoulos, GeorgiosBarnett, LionelMartyushev, L.M.Baroth, JulienMartyushev, LeonidBarr, Richard G.Marujo, LuisBarralon, PierreMaruta, KazukiBarrett, Adam B.Marzbanrad, FaezehBaruník, JozefMaschke, BernhardBasaran, CemalMasoumi, SamiraBashivan, PouyaMastriani, MarioBashkirtseva, IrinaMastroeni, L.Batalla, Jordi MongayMasuda, NaokiBaumert, MathiasMata, Maria EugéniaBayomy, A. MMatei, AniBechinger, ClemensMateo-Lázaro, JesúsBeckwith, Andrew WalcottMateos Roco, José MiguelBee, MarcoMatera, MauricioBeer, RandallMatilla-García, MarianoBeggs, John M.Matone, MarcoBehar, JoachimMatsoukas, ThemisBekiros, SteliosMatsumoto, RyutarohBellos, EvangelosMatsuta, TetsunaoBelman-Flores, J.M.Matúš, FrantišekBelonosko, AnatolyMaunder, RobBelsare, A. D.Mauri, RobertoBeltra, Rafael LahozMauro, JohnBenato, AlbertoMaybank, SteveBenatti, FabioMayer, ChristopherBenedetto, FrancescoMazzola, GuglielmoBengtsson, IngemarMcClure, JamesBenim, Ali CemalMcCulloch, MikeBen-Naim, AriehMcDonald, Kirk T.Benninger, DanielMcGregor, SimonBeranek, LadislavMcGuire, MichaelBerens, PhilippMcKenzie, EricaBeretta-Piccoli, MatteoMedved, VladimirBermudez, MarcelMeintanis, Simos G.Bernardi, GiulioMelis, RobertaBerthoumieu, YannickMelkikh, Alexey V.Berto, FilippoMeloni, CarloBertschinger, NilsMeloni, SimoneBesagni, GiorgioMelvin, William L.Beşdok, ErkanMendez, Martin OswaldoBeyerer, JürgenMéndez, VicençBhattacharia, SanjoyMenendez, HectorBhotto, Md. Zulfiquar AliMenéndez, Héctor D.Biagetti, GiorgioMeo, MariannaBialecka-Florjańczyk, EwaMerabia, SamyBianchi, GiuseppeMerletti, RobertoBianchini, CosimoMesa Sanchez, Oscar JoseBianco, VincenzoMestdag, TomBianucci, MarcoMetcalfe, StanBienertová-Vašků, JulieMezei, JózsefBiondo, Alessio EmanueleMichailidis, PanagiotisBird, SimeonMichalski, RadosławBishop, MarkMichel, FlorentBisi, MarziaMichieletto, DavideBisio, AlessandroMihailovic, DragutinBiswas, SoumyajyotiMilani, SimoneBithas, Petros S.Miłkowski, MarcinBizarro, João P. S.Miller, LuisBlanche, Pierre-alexandreMilne, BruceBlasco, JorgeMilone, CandidaBlázquez-Salcedo, Jose LuisMilton, KimballBlecich, PaoloMimoso, José PedroBlencowe, MilesMinea, Alina AdrianaBloch, MatthieuMing, DengmingBloem, PeterMiozzo, MarcoBo, TanMiranda, Jose Garcia VivasBobbio, AndreaMiret-Artés, SalvadorBode, LeticiaMiro, ShorashBogaert, JanMishra, AshokBogdan, PiotrMiśkiewicz, JanuszBogner, KonradMitsche, DieterBoguniewicz, JoannaMobed, NaderBondarescu, RuxandraMohajeri, NahidBopp, FritzMohammad-Djafari, AliBordallo, Heloisa N.Mohler, M. JaneBorgonovi, FaustoMohssen, MagdyBoriga, RaduMolinaroli, LucaBormashenko, EdwardMolinero, XavierBorodich, Feodor M.Monaco, VinnieBorovcnik, ManfredMondejar, Maria E.Bosiljka, TadicMondelli, MarcoBoškoski, PavleMontanari, Gian CarloBossomaier, TerryMontesi, DaniloBoström, KimMontessori, AndreaBosyk, Gustavo MartinMontufar, Guido F.Bottazzi, GiulioMoon, JongsubBoubchir, LarbiMora, HiginioBoucheron, StéphaneMora, ThierryBougheas, SpirosMorabito, Francesco CarloBourgine, PaulMorales Luna, GuillermoBourguignon, MarceloMoral-Muñoz, José AntonioBousia, AlexandraMoreno Arboleda, Francisco JavierBoyadzhiev, Khristo N.Moretto, EnricoBoyom, MichelMorgenthaler, StephanBoyom, Michel NguiffoMorison, GordonBozdogan, HamparsumMorley, BruceBrandner, KayMoroni, MonicaBranicki, MichalMorosuk, TatianaBraun, JamesMoroz, AdamBravetti, AlessandroMorrone, BiagioBrelsford, ChristaMorrone, PietropaoloBresme, FernandoMoshou, DimitriosBrigham, KatharineMota, AlziraBrini, EmilianoMountrouidou, XeniaBroadbridge, PhilipMousa, AmrBrodie, Ian M.Moustakidis, CharalamposBrody, JustinMück, WolfgangBross, ShragaMueller, MarkusBrouers, FrancoisMuga, J. GonzaloBrowne, DanMukherjee, Partha SarathiBrunton, Bingni WenMukherjee, PritamBruschi, David EdwardMukherjee, SayanBub, JeffreyMulder, JorisBuchmeister, BorutMüller-Vahl, Kirsten R.Bui, NicolaMungan, Carl E.Bukal, MarioMuñoz, José J.Bukkapatnam, Satish T.S.Murtagh, FionnBurini, DilettaMusa, Sarhan M.Burtini, GiuseppeMuscato, OrazioBuza, KrisztianMusgrave, IanCabboi, AlessandroMusslimani, ZiadCabral, João M. G.Myers, ChrisCabral, PedroMyong, R.S.Cabrerizo, Francisco JavierNabity, JamesCacciatori, Sergio L.Nadin, MihaiCafaro, CarloNagarajan, RaghavanCai, Yi-FuNaik, GaneshCaiado, CamilaNakamura, Tadas K.Cailliez, FabienNakao, HiroyaCaire, GiuseppeNalepa, JakubCaleffi, MarcelloNanni, LorisCallico, Gustavo M.Napoli, AlessandroCalvo Hernandez, ANapolitano, FrancescoCamarena-Martinez, DavidNaranjo, LizbethCampo Vázquez, CelesteNarnhofer, HeideCampo, Adrià TausteNarusawa, UichiroCano, AlbertoNassar, AntonioCanovas Pena, Jose S.Nasser, RajaiCao, BingyangNasuto, SlawomirCao, JindeNaudts, JanCapecci, ElisaNavara, MirkoCaporale, Guglielmo MariaNavarro-Mesa, Juan L.Caprara Vivoli, ValentinaNelson, Kenric P.Caraiani, PetreNemś, MagdalenaCaramés, Tiago FernándezNeri, IzaakCaravelli, FrancescoNestorovich, EkaterinaCarbia Carril, JoséNetočný, KarelCarbone, AnnaNewell, KarlCardoso, JaimeNg, Derrick Wing KwanCarlini, MaurizioNg, JasonCarminati, Maria ChiaraNguyen, NhaCarmona, Jose ManuelNian, JunCarpena, PedroNicolaou, NicolettaCarpi, LauraNicolis, GrégoireCarsteanu, Alin AndreiNielsen, HeberCarter, AlanNieto, Juan J.Carvalho, André DeNigmatullin, RamilCarvalho, LeonorNijsure, YogeshCarvalho, PauloNikas, George K.Casalino, GabriellaNikkhah-Moshaie, RoozbehCasarin, RobertoNikolaev, AlexanderCastellano, Javier GarcíaNikulov, AlexeyCastellano, Nicola GiuseppeNino-Ruiz, Elias D.Castiglioni, PaoloNiro, AlfonsoCastillo Sequera, José LuisNissen, IvorCatarino, IsabelNiven, RobertCattani, CarloNivre, JoakimCavalcante, Charles CasimiroNock, RichardCavalcanti, Eric G.Noh, GosanCejnar, PavelNoh, Jae DongCelesti, AntonioNojiri, Shin'ichiCembranos, J. A. R.Nolfi, StefanoCencini, MassimoNosonovsky, MichaelCerbino, RobertoNovitsky, Denis VCerello, PiergiorgioNurzaman, SuryaCerulo, LuigiOblak, DanielChacón-Acosta, GuillermoObuchi, TomoyukiChai, RifaiOcłoń, PawelChakraborty, BimanOdgaard, Peter FoghChalishajar, DimplekumarOdintsov, SergeiChamberlin, RalphOdqvist, JoakimChan, ChungOechtering, TobiasChan, FelixOh, SimonChander, HarishOikonomou, V.K.Chang, Lo-BinOizumi, MasafumiChang-Jian, Cai-WanOjovan, MichaelChaplain, MarkOkon, EliasCharalampos, PitasOlbrich, EckehardCharles-Cadogan, GodfreyOliveira Mimoso, José PedroChathoth, Suresh MavilaOliveira, Elismar R.Chau, Kwok-wingOliveira, Santiago Del RioChechkin, AlekseiOliveto, GiuseppeChen, BadongOlmos, Pablo M.Chen, BinqiangOlsen, Lars Ole RonnowChen, Bor-SenOmachi, ShinichiroChen, BoxingOneto, LucaChen, Chun-ChiOnken, ArnoChen, Der-chinOprea, IulianaChen, HongshuOrcioni, SimoneChen, I-TeOrciuoli, FrancescoChen, JianyongOrdonez, GonzaloChen, LingenOreshkov, OgnyanChen, LuOrtega, Guillermo JChen, Mu-SongOrtiz De Zárate, José M.Chen, Shyi-MingOrtiz-García, MarioChen, Ting-liO'Shaughnessy, DouglasChen, WenxiOssadtchi, AlexeyChen, XiOstoja-Starzewski, MartinChen, YanguangOświęcimka, PawełChen, YanlingOszust, MariuszChen, YuhuaOu, CongjieChen, ZonghaiOuerdane, HenniCheng, ChangqingOuldridge, ThomasCheng, ZhiyongOvchinnikov, IgorChernodub, MaximOzel, OmurChesneau, XavierPaez-Hernandez, RicardoChew, LockyuePahle, JürgenChi, SienPaja, WieslawChiachio, ManuelPalma, G.MassimoChiarelli, PieroPalyulin, Vladimir VladimirovichChiatti, LeonardoPanagiotidis, TheodoreChibbaro, SergioPanagiotopoulos, AlexandrosChicharro, D.Pandey, M.D.Chiementin, XavierPang, XiaodanChien, Feng-TsunPankratov, AndreyChih, MingchangPantaleo, AntonioChmiel, AnnaPantaleo, Antonio MarcoCho, Hwan-GuePantazis, YannisCho, Joon HoPanzeri, StefanoChodera, John D.Pap, GyulaChoi, Jun-WonPapathanasiou, JasonChoo, RaymondPappalardo, LucaChotorlishvili, LevanPappu, ChandraChristen, ThomasParadisi, PaoloChristodoulides, PaulPark, Choon-SuChu, YongbinPark, HyunggyuChuang, MichaelPark, NohhyunChui, Kwok TaiParra, Ruben D.Chun, Kwok PanParvan, Alexandru S.Ciaccio, Edward J.Passarella, FrancescaCiaramella, AngeloPasson, OliverCiuonzo, DomenicoPastore, RaffaeleClaeskens, GerdaPastorello, DavideClemente, RiccardoPaternostro, MauroCleuren, BartPatrascu, AndreiClisby, NathanPaunkovic, NikolaCluni, FedericoPavelka, MichalCockshott, WilliamPavlos, GeorgeCodetta-Raiteri, DanielePavsic, MatejCoecke, BobPaya, LuisCohen, EliahuPayaró, MiquelColangelo, GianpieroPazos, AlejandroColes, PatrickPearle, PhilipColonius, FritzPecht, Michael GerardComes, CalinPei, ZongruiComez, LuciaPeinke, JoachimComo, GiacomoPejaś, JerzyCongedo, Paolo MariaPeláez-Moreno, CarmenConnor, RichardPelorosso, RaffaeileConsoulas, ChristosPendrill, LeslieContreras-Reyes, Javier E.Peng, LinyuCooke, Thomas J.Peng, WeiwenCopot, CosminPennini, F.Corani, GiorgioPennisi, SebastianoCoro, GianpaoloPerarnau-Llobet, MartíCorominas-Murtra, BernatPerc, MatjazCortés López, Juan CarlosPerdigão, Rui A. P.Cosma, GeorginaPereira, MárioCosta, Cesar DaPeretti, Christian DeCosta, MárioPérez, Agustın-AgüeraCosta-jussà, Marta R.Pérez-Madrid, A.Covelli, CarminePeris, BernardoCraus, MiticǎPerivolaropoulos, LeandrosCrepeau, JohnPerkins, TheodoreCrippa, PaoloPerlick, VolkerCristelli, MatthieuPerram, John WilliamCroca, José Nunes RamalhoPerron, AurelienCroke, SarahPeshkov, IlyaCrokidakis, NunoPesta, MichalCrosignani, BrunoPetrellis, NikosCruz-Hernández, CésarPétritis, DimitriCudennec, ChristophePhinyomark, AngkoonCuesta-Frau, DavidPiantadosi, Steven T.Cui, XingranPiasecki, KrzysztofCummins, FredPichl, LukasCurado, Evaldo M. F.Pietronero, LucianoCutler, AdelePimenta Dinis, Maria AlziraCzinner, Viktor G.Pinho, Armando J.Da Luz, Marcos Gomes EleutérioPinoli, Jean-CharlesDa Silva, Mario MarquesPinto, CarlaD'Abramo, GermanoPiovani, DuccioDagdug, LeonardoPires, CesaltinaDai, WeihuiPirner, Hans JürgenDaivis, Peter J.Piskorski, JarosławD'Andreagiovanni, FabioPistoia, FrancescaDanner, Simon MPivoluska, MatejDantcheva, AntitzaPlakandaras, VasiliosDappiaggi, ClaudioPlanat, MichelDariusz, WrzesińskiPlank, Michael J.Darvishi Kamachali, RezaPlastina, FrancescoDas, ManoharPlastino, AngelDassios, IoannisPlastino, AngeloDassios, Ioannis K.Platt, TrevorDatcheva, MariaPławiak, PawełDatta, JyotishkaPleimling, MichelDaum, FredPlesch, MartinDavid, SergioPoelzing, StevenDavila-Velderrain, JosePoet, RonDavis, SergioPogrebnjak, AlexanderDaw, StuartPohl, ChristineDawid, PhilipPokrajac, DavidDe Andrade Alves, Luiz GustavoPoletti, DarioDe Araujo, Jose CarlosPolettini, MatteoDe Arcangelis, LucillaPolimeno, AntoninoDe Bragança Pereira, Carlos AlbertoPoliti, AntonioDe Brevern, AlexandrePompe, BernardDe Capitani, LucioPoncet, SébastienDe Castro, MarioPooranian, ZahraDe Chiara, GabrielePop, IoanDe Giuli, Maria ElenaPopescu, Mihail N.De Jong, WilPorporato, AmilcareDe Kerret, PaulPorta, AlbertoDe Leon, ManuelPost, ThierryDe Meo, PasqualePoti, ValerioDe Miras, Juan RuizPotirakis, SteliosDe Oliveira Júnior, SilvioPoveda, GermanDe Pascale, AndreaPovstenko, YuriyDe Pascali, ChiaraPradas, MarcDe Rosa, SergioPrasath, V. B. SuryaDebeir, OlivierPrażmowska, MałgorzataDebowski, LukaszPrażmowski, KrzysztofDębski, AdamPrehl, JanettDeDeo, SimonPressé, SteveDegaetano-Ortlieb, StefaniaPreziosi, ValentinaDehmer, MatthiasPriesemann, ViolaDeiters, UlrichProesmans, KarelDel Campo, AdolfoProtzner, Andrea B.Delgado, ManuelPruessner, GunnarDella Rossa, FabioPsannis, KonstantinosDelogu, FrancescoPuerta Callejón, José MiguelDel-Val-Noguera, ElenaPulido, InmaculadaDeMazumder, DeeptankarQiao, YuansongDemetrius, LloydQin, SanboDeng, JeremiahQualls, Whitney A.Deng, MingcongQuarati, PieroDennard, LindaQuax, RickDesbois, DominiqueQueiros, Silvio M. DuarteDesmet, BernardQuintero-Quiroz, CarlosDette, HolgerQuitadamo, LuciaDeufemia, VincenzoRaboso, MarianoDewan, AshrafRadac, Mircea-BogdanDi Gravio, GiulioRadak, BrianDi Crescenzo, AntonioRaddant, MatthiasDi Francescomarino, ChiaraRadenković, SlavkoDi Martino, FerdinandoRadica, GojmirDi Nola, AntonioRadulovic, JovanaDi Vita, AndreaRafał, Marcin LaskowskiDi Zio, SimoneRagulskis, MinvydasDieks, DennisRahnejat, HomerDienes, ZoltanRajendra Acharya, U.Diep, Hung T.Rajsekhar, DeepthiDijkstra, Bauke W.Rakhshan, AliDima, Mihai-OctavianRamachandran, K. I.Dimitriou, DimitriosRamampiaro, HeriDimitrov, Alexander G.Ramirez, Israel ReyesDinis, LuisRamírez-Reyes, AbdielDinneen, Michael JRamos, Célia M. Q.Dionisio, AndreiaRamos, Rubens VianaDivigalipitiya, PrasannaRamsey, Vincent K.Dixit, Purushottam D.Ran, ShijuDobrovolny, Hana M.Rapaport, WilliamDodd, Ian C.Rashidi, Mohmad MehdiDodig-Crnkovic, GordanaRashkovits, RamiDolan, BrianRathgeber, AndreasDolfin, MarinaRathie, PushpaDomingues, PedroRaudino, AntonioDong, BoxiangRauh, JohannesDong, DaoyiRavelli, FlaviaDong, YiqiuRavelli, SilviaDonner, ReikRavi, AnanthDoran, DerekRaz, OrenDove, GuyReeke, George N.Dreibholz, ThomasReichardt, ChristianDressel, JustinReichhardt, CharlesDreżewski, RafałReis, Pedro Miguel RamosDuan, JinmingRen, JinchangDuann, Jeng-Ren (J.R.)Renevey, PhilippeDuarte-Mermoud, Manuel A.Rensburg, E.J. Janse VanDubenko, IgorRequardt, ManfredDudczyk, JanuszResinas, ManuelDukka, KCRétvári, GáborDumont, RemiRevell, AlistairDungey, MardiReyes, José SantosDuong, Quang TrungReyzin, LevDupuis, FrédéricRheem, JaeyeolDutton, ZacharyRhudy, MatthewDwivedi, DipankarRibeiro, HaroldoDworak, PawełRicciuti, CostantinoDziarmaga, JacekRichman, JoshuaEbaid, AbdelhalimRichmond, Christ D.Eddy, BrianRichter, HendrikEdiriweera, SisiraRichter, Wolf-dieterEgolf, Peter W.Ridgway, SamEibenberger, SandraRidolfi, ElenaEisert, JensRiechers, Paul M.Eleuch, HichemRiechert, UweElfadaly, FadlallaRiihonen, TaneliEllahi (R. Ellahi), RahmatRinaldo, RobertoEllerma, DavidRingbauer, MartinElMoslimany, AhmadRio, CarlosElsalloukh, HassanRios, MiguelEl-Shahat, AdelRiquelme, AdriánElsinger, HelmutRisse, MarianEltayeb, Mohammed ERitort, FèlixElwakil, Ahmed S.Rivero, Cristian RodriguezElze, Hans-ThomasRodger, James A.Emerson, JosephRodrigues, Alexandre A. P.Engelbrecht, KurtRodrigues, JoãoEnßlin, TorstenRodrigues, Maria Manuela FernandesEntin-Wohlmann, OraRodriguez, Jose M.Escassut, AlainRodríguez, Juan J.Escudero, JavierRodriguez, LeoEsfahanian, MahdiRodríguez, Rosa M.Espinosa, MariaRoe, Daniel R.Esposito, MassimilianoRoffo, GiorgioEsquivel, Rodolfo O.Roger, SandraEssafi, KarimRogers, DavidEstellé, PatriceRogosin, SergeiEstrada, ErnestoRoldan, EdgarEvans, MartinRoldán, ÉdgarEvans, MichaelRomagnoli, Pierre PaulExpert, PaulRomano, ElviraFaber, ManfriedRomera, ElviraFaes, LucaRomero-Troncoso, Rene De JesusFaggini, MarisaRominger, Andrew J.Falcucci, GiacomoRooh Khurram, RoohFalniowski, FryderykRosas, FernandoFan, PingyiRosić, BojanaFan, WenkeRossi, LucaFang, YinfengRosu, HaretFarrell, MaxRoterman, IrenaFasano, MatteoRoth, Bradley J.Federolf, PeterRothkrantz, LeonFedorov, AlekseyRotter, IngridFei, Cheng-weiRotundo, GiuliaFeidt, MichelRoumy, AlineFelice, DomenicoRoussey, CatherineFelisberto, PauloRovira, AntonioFellouris, GeorgiosRoy, CerquetiFeng, WanpengRoy, Raphaëlle N.Feng, YuRozakis, SteliosFeng, YunlongRubi, MiguelFereidountabar, AmirhosseinRubi, Miguel J.Ferracuti, FrancescoRubio, F. JavierFerrando, RiccardoRucco, MatteoFerrara, MassimilianoRuddell, BenjaminFerraro, AlessandroRuess, JakobFerreira, Eduarda PintoRuiz, L. M. SanchesFerrero, AlessandroRuiz, Magda L.Ferro, MarcelloRuss, JohnFiedor, PawełRuziboev, MarksFigliola, AlejandraRwebangira, Mugizi RobertFiglus, TomaszSabar, NasserFigueiras, EditeSabín, CarlosFilatrella, GiovanniSáchez Soto, Luis LorenzoFillion-Gourdeau, FrançoisSadakane, KunihikoFink, GabrielSaez-Gomez, DiegoFinn, ConorSafaai, HoumanFiorentino, MauroSaffari Pour, MohsenFiori, SimoneSagawa, TakahiroFischer, Franz DieterSaha, PriyaFischer, Matthias J.Said, SalemFistola, RomanoSaitoh, TakeshiFlorea, Olivia AnaSajeed, ShihanFong, Silas L.Salinas, Hugo S.Forbes, Angus G.Samadani, EhsanForestier, GermainSamani, AfshinFortuna, LuigiSamek, WojciechFosgerau, MogensSan Miguel, AdrianaFotouhi, AbbasSanchez, DavidFouquier D'Hérouël, AymericSanchez-Salas, NormaFradin, CécileSanchez-Taltavull, DanielFrancisco, Caamaño IsornaSandev, TrifceFrank, SSandoval Orozco, Ana LucilaFränti, PasiSandoval, LeonidasFranzosi, RobertoSands, DavidFreddi, AlessandroSanei, SaeidFreudenberger, JürgenSantos, José JoaquimFriedrich, TobiasSantos, Laurita DosFriston, KarlSantra, SiddharthaFroese, AnnaSanz, Angel S.Fronsdal, ChristianSapora, AlbertoFu, ZuntaoSaravanakumar, RamasamyFujisawa, HironoriSarbu, IoanFujiwara, YoshiSardina, GaetanoFulginei, Francesco RigantiSarkisyan-Grinbaum, EdwardFullér, RobertSarlis, Prof. NFung, ThomasSarracino, AlessandroFurtula, BorisSarri, GianlucaGabora, LianeSarwate, Anand D.Gadhoumi, KaisSasa, Shin-ichiGadomski, AdamSauer, TilmanGadouleau, MaximilienScalo, CarloGagie, TravisScargle, JeffreyGajowniczek, KrzysztofSchaeffer, Satu ElisaGalam, SergeScharfenaker, EllisGalanis, GeorgeScheibelhofer, PeterGaleazzi, RobertaSchilbrack, KevinGalperin, MichaelSchilling, OlegGama Goicochea, ArmandoSchmandt, BastianGamo, JavierSchmidt, Christian AndrésGamsjäger, ErnstSchmidt, SabineGanguly, PritamSchneider, AntonGao, BinSchneider, RaimundGao, SongScholkmann, FelixGao, ZhongkeScholz, JohannesGarcía Díaz, PilarSchroeck, FranklinGarcia, Nuno M.Schumann, AndrewGarcia-Leon, ManuelSchwarz, GottfriedGardini, LauraScullion, TomGargiulo, GaetanoScurrell, MikeGarland, JoshuaSeghouane, KarimGaunt, RobertSelby, JohnGavrilov, MomčiloSemkov, KrumGAVRILUŢ, Alina CristianaSenthilnath, J.Gawron, PiotrSeoane, José A.Gay-Balmaz, FrancoisSergeyev, Yaroslav D.Gazeau, Jean PierreSergi, AlessandroGe, HaoSergio, CurilefGeiger, Bernhard C.Seshadreesan, KaushikGelbstein, YanivSessa, SalvatoreGelbwaser, DavidSeverns, Paul MGencaga, DenizShabgard, HamidrezaGençay, RamazanShagrir, OronGendron, PaulShahzad, JawadGenes, ClaudiuShanmugam, RamGeng, RuiliShao, LijingGentili, ClaudioShapiro, Jeffrey H.Georgeon, OlivierSharaev, MaksimGeronimo, Andrew M.Sharpee, TatyanaGervino, G.Shawny, Xiao XiaoGeurdes, HanShe, XiaohuiGharabaghi, BahramSheikholeslami, Seyed MahmoudGhazi-Zahedi, KeyanSheng, WenyiGholamalizadeh, EhsanSherbakov, SergeiGhoraani, BehnazSheremet, MikhailGhosh, AbhikSheremet, Mikhail AleksandrovichGhosh, ArnabShi, YongtangGiannakis, KonstantinosShikano, YutakaGiannerini, SimoneShinohara, Russell TakeshiGiannoccaro, IlariaShiskin, SergeiGibson, Edward (Ted)Shum, KennethGielen, SteffenSidorov, Denis N.Gil, DavidSiebenhühner, FelixGilbert, RobertSienkiewicz-Małyjurek, KatarzynaGill, RichardSiettos, ConstantinosGilli, ManfredSignorini, Maria GabriellaGillich, Gilbert-RainerSilva Junior, RaimundoGiménez, Raimundo RealSilva, Cristiana J.Gingrich, ToddSilva, Luiz Eduardo VirgilioGinsca, Alexandru LucianSilva, Maria EduardaGioia, AndreaSilva, Thiago HenriqueGiorgi, Gian LucaSimani, SilvioGiuliani, AlessandroSimon, JoanGlavatskiy, KirillSimon, SergiGlimm, TilmannSimonetti, BiagioGlowacz, AdamSimpson, GideonGnatyuk, SergiySiuly, SiulyGodang, RomulusSivasundaram, SeenithGodinez Tello, RichardSiwy, ZuzannaGogioso, StefanoSkardal, Per SebastianGokaraju, BalakrishnaSkeppstedt, MariaGolan, AmosSkilling, JohnGoldenbaum, MarioSkotiniotis, MichalisGoldstein, SheldonSkupin, PiotrGolubev, DmitrySlagmolen, Bram J.J.Gomez, Ignacio SebastiánSlijepcevic, SinisaGomez-Barrero, MartaSlomczynski, WojciechGómez-Déniz, EmilioSmall, MichaelGomez-Pilar, JavierSmart, PaulGompper, GerhardŚmieja, MarekGonçalves, HernâniSmirne, AndreaGonçalves, Paulo Jorge SequeiraSmith, Edward R.Gong, MeiSmith, StefanGontis, VygintasSmolić, IvicaGonzalez, FernandoSohl, TerryGonzalez-Ayala, JulianSole, PatrickGonzález-de-la-Rosa, Juan-JoséSolis, FranciscoGonzález-Díaz, HumbertoSommer, StefanGonzález-Rubio, JesúsSon, Seung-WooGonzalo-Martín, ConsueloSong, HoubingGoodarzi, Farhad A.Sookhak, MehdiGoodheart, BenjaminSorguven, EsraGoodwell, AllisonSothmann, BjörnGordon, JeffreySoundarajan, SuchetaGórecki, TomaszSousa Fragoso, RuiGórski, MarcinSouto, AndréGorte, BenSpagnolo, NicolòGosak, MarkoSparacino, GiovanniGoto, Shin-ItiroSparavigna, Amelia CarolinaGough, JohnSpekkink, WouterGoupil, ChristopheSpiliotis, MikeGoussis, DimitrisSpina, DamianoGoyal, ManiaSpohn, HerbertGoyeneche, DardoSpüler, MartinGradojevic, NikolaSquartini, TizianoGralla, Samuel E.Srikanth, R.Gramacy, Robert B.Srinivasa, ArunGramaglia, MarcoStacey, BlakeGrazian, ClaraStamatatos, EfstathiosGreen, JasonStamova, IvankaGreen, MichaelStanciu, CameliaGrilli, JacopoStarosolski, RomanGrimaldi, DomenicoStavrou, Photios A.Grimaldi, SalvatoreŠtefaňák, MartinGrinbaum, AlexeiStefanatos, DionisisGrøn, ØyvindStemler, ThomasGross, ThiloStephan, Klaas E.Groza, BogdanStewart, Terrence C.Gu, YinaStill, SusanneGuariglia, EmanuelStojkovic, DejanGuarnieri, GiacomoStoltz, GabrielGudowska-Nowak, EwaStramaglia, SebastianoGuendelman, EduardoStrapasson, João E.Guenza, MarinaStratton, PeterGuirao, Juan Luis GarcíaStrozzi, FernandaGujarati, PurushottamStrumiłło, PawełGullo, ParideStrzalka, DominikGuney, DurduStutzer, MichaelGunkel, David JStyle, RobertGuntuboyina, AdityaSu, JianxiGuo, YuzhuSuarez, AlvaroGurjeet, DhesiSuarez, ElisabetGutiérrez Tobal, Gonzalo CésarSubasi, AbdulhamitGutiérrez-Tobal, Gonzalo C.Subiantoro, AlisonGuttmann, Anthony (Tony)Sudarsky, DanielGuyeux, ChristopheSuhov, YuriGuzowska, Malgorzata KlaudiaSukharev, SergeiHaack, GéraldineSułowicz, MaciejHaba, ZbigniewSumelka, WojciechHagerstrom, AaronSun, AlexanderHale, JackSun, HuafeiHall, Michael J WSun, Jia RuiHamhalter, JanSun, JiangwenHampel, DavidSun, KeHamza, BenSun, LiHan, SunghyuSun, ShiliangHanasaki, ItsuoSurana, K.S.Hangos, KatalinSuyari, HirokiHannemann-Tamas, RalfSuzuki, HiroyukiHannig, JanSuzuki, ReijiHaq, Rizwan UlSvoboda, J.Haramaty, EladSvozil, KarlHarandi, Mehrtash T.Swendsen, RobertHardal, Ali Ümit CemalSyczewska, MalgorzataHarish, GargSyed, Junaid NawazHarko, TiberiuSzabadics, JánosHarmandaris, VagelisSzabo, GyorgyHarre, MichaelSzabo, LorandHarremoës, PeterSzavits-Nossan, JurajHarrison, WillieSzczepanski, JanuszHarshman, NathanSze, Raymond Nung-SingHarte, JohnSzederkényi, GáborHartich, DavidSzékely, GáborHartmann, CarstenSzigeti, StuartHaruna, TaichiSzkaliczki, TiborHassan, Mohamed AhmedTadmor, RafaelHassani, HamedTakahashi, DanielHATANO, NaomichiTakahashi, TatsujiHatziadoniu, ConstantineTakayasu, HidekiHaug-Warberg, ToreTal, IdoHauke, PhilippTalakoub, OmidHaw, Jing-YanTamura, RyoHawton, MargaretTanahashi, NorihiroHayajneh, Khaled F.Tang, HongHayakawa, HisaoTang, LeileiHayakawa, MasashiTang, YinHayashida, MorihiroTaniguchi, ShigeruHeijungs, ReinoutTanimoto, JunHellings, ChristophTar, JozsefHemakom, ApitTărniceriu, DanielaHen, ItayTartaglia, AngeloHenine, HocineTatarinova, Tatiana V.Hennessy, DavidTaufer, EmanueleHerdrich, GeorgTchatoka, Firmin DokoHernández-González, JerónimoTelesca, LucianoHernández-Lobato, José MiguelTemko, AndriyHerrmann, J. MichaelTepljakov, AlekseiHerrmann, KlausTervonen, Jouni K.Herwig, HeinzTheil, FlorianHess, PaulTheis, Dirk OliverHettiarachchi, Imali ThanujaThiruvengadam, MageshHeydecker, BenjaminTilloy, AntoineHiblot, JulienTimme, NicholasHickinbotham, SimonTkachenko, DenisHidalgo, ArturoTlelo-Cuautle, EstebanHidetoshi, KonnoToda, Alexis AkiraHilbert, MartinTodd, MikeHilbert, StefanToffano, ZenoHirata, YoshitoToffoli, TommasoHiziroglu, HuseyinTohme, FernandoHjorth, PederTokuhiro, AkiraHofmann, RalfTomar, Vikrant SinghHölbl, MarkoTononi, GiulioHolik, Federico HernánTorabi, MohsenHong, Song-NamToral, AntonioHong, Wei-ChiangTorrens, PaulHornung, GrégoireTorrieri, GiorgioHorsch, MartinToussaint, Udo VonHorton, W. TravisTramontana, FabioHorvát, Emőke-ÁgnesTrancossi, MicheleHorvatić, DavorTrapin, LucaHossenfelder, SabineTravieso-Gonzalez, Carlos M.Hou, Shuhn-ShyurngTravlos, IliasHovorka, OndrejTrevathan, JarrodHron, KarelTrevizoli, Paulo ViniciusHsiao, Hsu-ChunTriantaphyllou, EvangelosHsiao, Kai-LongTrifonov, Peter V.Hsiao, Shen-FuTrivellato, BarbaraHsu, Li-ChiTrovati, MarcelloHu, Bei LokTsai, Chih-FongHu, Ya-PengTsai, Chun-WeiHuang, GordonTsai, Yao-HongHuang, Hen-HsenTscharner, Vincent VonHuang, JunyiTse, Ka KuiHuang, KejunTsinos, Christos G.Huang, Shih-FengTsonis, AnastasioHuang, ShoudongTsyganova, Julia V.Huang, WenTuck, AdrianHubbart, JasonTuckerman, MarkHuber, MarcoTura, JordiHuber, MarcusTurkanović, MuhamedHughes, Keith H.Turki, SadokHumeau-Heurtier, AnneTurner, BrandonHuminic, AngelTurner, MarkHuminic, GabrielaTurocy, TheodoreHung, HungTušek, JakaHung, Ling-YanUddin, Gazi SalahHwang, Chi-HungUeshima, NobufumiHwang, GisukUffink, JosIbl, MartinUllah, ShatifIckowicz, AdrienUnakafov, Anton M.Igasaki, TomohikoUnakafova, ValentinaIglesias, José RobertoUohashi, KeikoIkki, SalamaUrniezius, RenaldasIliyasu, Abdullah M.Uruba, VáclavInce, RobinUshizima, DanielaIngber, LesterVaidogas, Egidijus RytasInsausti, XabierVaitkus, AndriusInuso, GiuseppinaValdinoci, EnricoIpšić, IvoValencia, José F.Isidro, Jose M.Valenti, DavideIslam, SyedValenti, Vitor EngraciaIto, SosukeValentino, Eleonora DiIzquierdo, Eduardo J.Vallino, JosephIzquierdo, Segismundo S.Valverde-Albacete, Francisco JIzumida, YukiValyrakis, ManousosJack, Rachael E.Vamvoudakis, KyriakosJack, RobertVan Huffel, SabineJacob, Seibu MaryVan Maldeghem, HendrikJacobi, IanVan Putten, Michel J.A.M.Jacobs, DonaldVan Someren, MaartenJacquir, SabirVan Vliet, MarijnJaeger, GreggVan Wijland, FredJain, AkankshaVanapalli, SrinivasJakus, DamirVandaele, Nico J.James, RyanVargas-Rosales, CésarJang, Gun HoVarshney, Kush R.Jasra, AjayVassiliev, DmitriJauregui, MaxVazquez-Vilar, GonzaloJavarone, Marco AlbertoVedula, PrakashJeleń, LukaszVelazquez, LuisberisJenčová, AnnaVélez Upegui, Jorge JuliánJendrzejczyk-Handzlik, DominikaVenegas, MariaJenkins, KennethVerda, VittorioJi, MingyueVerdoolaege, GeertJi, SungchulVergara, J. DavidJiang, HaoVernieri, DanieleJiang, JingfengVersteegh, Marijn A. M.Jiang, LanlanVesin, Jean-MarcJiang, YizhangVianna, Reinaldo OliveiraJin, ZhigangVicente, RaulJog, VarunVickers, StevenJohnson, Sarah JVictor, JonathanJolley, Caroline J.Vignudelli, StefanoJones, ChrisVila, Xosé A.Jones, J CliffVilar, JoseJong, Gwo-jiaVillani, MarcoJonsdottir, JohannaVillarroel, Grover ZuritaJorgensen, PalleVillecco, FrancescoJorgensen, Palle E.T.Vilmart, GillesJoseph, JojoVirosztek, DánielJoshi, SiddarthVisentin, DenisJoutsijoki, HenryVitabile, SalvatoreJovanovic, FranckVitanov, NikolayJuárez, Isaac Leobardo SánchezVitanov, Nikolay V.Juba, BrendanVitányi, PaulJung, Young-DaeVoit, EberhardJurado, Juan GarridoVolkov, Alexey N.Jurkiewicz, JerzyVolosniev, A. G.Jurman, GiuseppeVon Spakovsky, MichaelKahirdeh, AliVon Toussaint, UdoKakar, Muhammad RafiqVontobel, Pascal OKalinay, PavolVorovencii, IosifKalogeropoulos, NikosVorwerk, JohannesKamidis, NikolaosVucelja, MarijaKamiński, MarcinVucetich, HéctorKang, Dae-KiVullings, RikKang, KyongokVulpe, AlexandruKang, WeiVulpiani, AngeloKaniadakis, GiorgioWacławczyk, MartaKantarcioglu, MuratWada, TatsuakiKantardzic, Mehmed M.Wagoner, JasonKaragrigoriou, AlexWalk, NathanKarakatsanis, L.P.Wallace, DavidKarami, AzamWan, FengKarasuyama, MasayukiWang, AnzhongKarimi, HamidWang, DengKarimi, NaderWang, DongKarlgaard, Christopher D.Wang, JinfengKarlin, IlyaWang, LigongKarperien, Audrey L.Wang, LingKarpov, EvgueniWang, Lin-ShuKarsai, IstvanWang, MeiKaryotis, VasileiosWang, ShibingKastner, RuthWang, ShuangfengKateris, DimitriosWang, WenwuKatkov, MikhailWang, XiaofangKatsura, HoshoWang, YangKatunin, AndrzejWartena, ChristianKauranne, TuomoWatanabe, EiichiKavic, MichaelWatanabe, KazuhoKawalec, AdamWaterstraat, NilsKawazura, YoheiWay, Michael J.Kay, JimWeber, StefanKazakis, NerantzisWedemann, Roseli S.Ke, Jia-HongWeijs, StevenKelbert, MarkWeinert, FriedelKeller, KarstenWernik, JacekKelly, James F.West, Bruce J.Kenett, Dror Y.Westerbäck, ThomasKeogh, EamonnWharton, KenKeshavarzian, SajjadWhitaker, M.A.B. (Martin Andrew)Khalil, MohammadWhite, MartinKhalilia, MohammedWhite, RogerKhan, IlyasWibisono, AndreKhandoker, Ahsan HabibWibowo, SantosoKharrazi, AliWieczorek, PiotrKhlopov, Maxim Yu.Wiederholt, MirkoKhrennikov, AndreiWieland, ChristophKhurshudyan, MartirosWilk, GrzegorzKillick, RebeccaWilson, Christopher G.Kim, BoHungWilson, EddieKIM, CheonshikWinther, OleKim, Dong HanWolf, AlexKim, Eun-jinWolf, ChristianKim, HyunjuWolpert, DavidKim, Jennifer A.Wong, Ka-ChunKim, Jung TaeWong, LawrenceKim, SangdanWong, M.L. DennisKim, Sang-HyoWoo, Jae OhKimiaghalam, NavidWoodbury, Allan D.Kimovski, DragiWoodhead, ErikKinateder, HaraldWootters, WilliamKinoshita, TamotuWoźniak, MarcinKiverstein, JulianWrzesiński, DariuszKjelstrup, SigneWu, Hau-tiengKlados, ManousosWu, Jui-yuKleidis, KostasWu, Shuen-DeKlein, TonyWu, XiaoqunKlika, VáclavWu, XimingKlimenko, AlexanderXavier, AntonioKlimenko, Alexander Y.Xiao, BoqiKnuplesch, DavidXiao, LiangliangKnuth, KevinXiao, MingKo, Wen-ChangXie, HongboKoch, TobiasXu, DianguoKochanek, KrzysztofXu, HaiyanKochubei, AnatolyXu, MingtianKohar, VivekXu, ShuKolchinsky, ArtemyXu, XiaomingKolkiewicz, Adam W.Xu, ZelinKomabayashi, TetsuyaXue, XianwuKomiyama, JunpeiYablonsky, GregoryKong, WanzengYamada, ToruKononovicius, AleksejusYamagishi, MasaoKonstantas, DimitriYan, ZhiyuanKonstantinou, GeorgiosYang, FanKorbel, JanYang, Hyun JongKordos, MirosławYang, JasonKorzekwa, KamilYang, JianhuaKorzhik, ValeryYang, Min-HsiungKostina, VictoriaYang, ShengKostopoulos, SpirosYang, ShengtianKotsiantis, SotirisYang, ShimingKouchaki, SamanehYang, Tian-ZhiKoutsouris, DimitrisYang, YuanKowalski, Frank V.Yang, Zhi-XinKowalski, GregoryYano, KeisukeKrajewski, JarekYanushkevich, SvetlanaKrasnov, Oleg A.Yatsalo, Boris I.Kravtsov, SergeyYe, JunKremer, GilbertoYe, ZhishengKudryashov, Boris D.Yentes, Jenna M.Kuffer, MonikaYeo, HwasooKugiumtzis, DimitrisYetis, Cenk M.Kukreja, MuskanYi, Sang-HoonKulik, RafalYi, XinpingKumagai, AtsuyaYin, PenghangKumamoto, KazuoYing, MingshengKuperberg, GregYook, Soon-HyungKurabayashi, ShuichiYoon, Joon YongKurihara, SeijiYoon, Peter H.Kurniawan, ErnestYouk, HyunKuś, MarekYu, Hak KiKuzuoka, ShigeakiYu, KemingKwak, Ho-YoungYu, MeichenKwiecień, JoannaYu, ZeyunKyprianidis, I.M.Yuasa, KazuyaLa Manna, MarcoYue, YuanzhengLai, LifengYuen, ChauLaio, AlessandroYuen, Ka-VengLake, Douglas E.Yunusa-Kaltungo, AkiluLamata, LucasZagorecki, AdamLambrou, TryphonZagouras, AthanassiosLampart, MarekZahidi, KarimLand, MartinZahorian, Stephen A.Langer, AndreasZambrano, DavideLangley, PhilipZampieri, MarcosLaroche, BeatriceZanella, MattiaLarrarte, FrédériqueZangi, RonenLarson-Prior, Linda J.Zanin, MassimilianoLatella, IvanZavadskas, Edmundas KazimierasLaudisa, FedericoZbeeb, KhaledLaurent, MevelZboril, FrantisekLavasan, Arash AlimardaniZdunek, RafalLawyer, GlennZelevinsky, VladimirLazaro, JesusZema, Demetrio AntonioLe Clainche, SoledadZenil, HectorLê, Hông VânZhan, HaifeiLebrun, JaneZhang, BowuLedrappier, FrançoisZhang, HaochunLee, Eon SooZhang, LeLee, GihyounZhang, PanLee, Ming-TsangZhang, QiLee, Suk JinZhang, TonghuaLee, Tae H.Zhang, Wei-QiangLeff, Harvey SZhang, XiaoguangLefkidis, GeorgZhang, XieLehman, Joel AnthonyZhang, YangjunLeike, Reimar H.Zhang, YiLelea, DorinZhang, YuLemus, EnriqueZhang, YudongLenzi, Ervin KaminskiZhang, YushuLeo, MarcoZhang, ZhenjiangLeón, GabrielZhao, ChengLeone, AntonioZhao, HangboLeonelli, CristinaZhao, JichaoLeonski, WieslawZhou, Bi-ChengLerga, JonatanZhou, HuiyuLerma, ClaudiaZhou, LiangLeroux, CamilleZhou, SiyiLetelier, Juan CarlosZhu, LingjiongLeung, CarsonZhu, MingyaoLeyvraz, FrançoisZhu, ShunpengLezoray, OlivierZhu, YongLi, Chang-TsunZhu, YungangLi, DuanZieliński, WojciechLi, GuanchenZiemke, TomLi, HuaizhongZimmer, JohannesLi, PeterZmeskal, OldrichLi, QianqianZonta, FrancescoLi, RongjianZorzi, MattiaLI, RuiyingZosso, DominiqueLi, TianchengZotin, AlexeyLi, WentianZou, Yue-XianLi, XiangZubiaga, ArkaitzLi, XiaodongZunino, LucianoLi, XiaoliZusai, DaiLi, YingsongZylberberg, Joel

